# Green Pervious Concrete Containing Diatomaceous Earth as Supplementary Cementitous Materials for Pavement Applications

**DOI:** 10.3390/ma16010048

**Published:** 2022-12-21

**Authors:** Alexander Gladwin Alex, Prakash Arul Jose, Mohammad Saberian, Jie Li

**Affiliations:** 1 Department of Building Construction Technology, Technical Vocational Training Institute, Addis Ababa, Ethiopia; 2 Department of Civil Engineering, Paavai Engineering College, Namakkal 637 018, India; 3 School of Engineering, RMIT University, Melbourne, VIC 3000, Australia

**Keywords:** pervious concrete, diatomaceous earth, C-S-H gel, amorphous silica

## Abstract

Portland cement porous concrete (PCPC) has received immense interest recently due to its environmental aids. Its porous structure helps to reduce the water runoff amount while improving the recharge of groundwater. Earlier studies have concentrated on illustrating and knowing the functional as well as structural properties of PCPC. However, very few studies are available on PCPC in combination with natural silica sources as supplementary cementitious materials (SCMs). Most SCMs are by-products of industrial manufacturing processes and cause some environmental concerns, but with their pozzolanic effect, they could be utilized as partial substitute materials for ordinary Portland cement (OPC) to enhance the strength as well as durability performance. The aim of this study is to evaluate the effects of diatomaceous earth (DE) as a supplementary cementitious material for partial substitution of OPC for Portland cement porous concrete application. Compression strength, split tensile strength, and flexural strength tests were performed to determine the effect of partial replacement. To investigate the impact of test variables, basic tests, including void content and water permeability, were also performed. Compared to the control concrete, the results show that a 15% replacement of cement with DE significantly increased the compressive strength (by 53%) while also providing adequate porosity and better water permeability. Statistical analysis (ANOVA) and regression analysis showed that there is a significant (*p* < 0.05) growth within the physical characteristics of concrete upon the replacement of cement by 15% DE. Collectively, the replacement of cement with DE could not only improve the concrete strength but also reduce the consumption of cement, thereby lessening the cost of construction as well as indirectly reducing the carbon footprint.

## 1. Introduction

Most of the places covered with impervious surfaces, such as concrete, due to urbanization, resulted in groundwater table reduction. Pervious concrete pavements, which are open-graded concrete with interconnecting pores with excellent water permeability and porosity, minimize the issue by allowing the seep-through of rain and stormwater into the ground [1,2]. The tendency of these structures to propagate water helps reduce the amount of stormwater runoff and recharges the groundwater [3,4,5]. Additionally, Portland cement pervious concrete (PCPC) decreases puddles on the road, exhibits sound absorption brought on by tire–pavement interaction, and enhances water quality through percolation [6,7,8]. In comparison to standard concrete, PCPC has a gradation with fewer or without fine aggregates (4.75 mm), and whenever the gradation keeps changing, the porosity ranges from 15 to 35%, with lower strength qualities than standard concrete. As a result, PCPC pavements are concentrated on low-traffic highways, parking spaces, riding tracks, sidewalks, and rural roads, all of which make up a large component of the existing pavement in urban zones.

Cement, one of the most extensively utilized construction materials, has caused the emission of a considerable amount of CO_2_ into the atmosphere, which has an adverse effect on the global environment [9,10]. Mostly in the near future, such a rise in carbon emissions could make a significant contribution to unfavorable climatic changes. To minimize the negative impact on the environment as well as energy use, several supplementary cementitious materials (SCMs) as partial replacements for cement are continuously being developed [11,12,13,14]. The pozzolanic assets of SCMs cause the reaction with Ca(OH)_2_ (Calcium Hydroxide) and water to form C-S-H (Calcium-Silicate-Hydrates) [15], which is analogous to the C-S-H formed during the hydration process of cement. This further facilitates the efficient replacement of SCMs with cement in cementitious composites and also produces concrete with better long-term strength and durability properties. Focusing on the conservation of natural resources and environmental safety, several studies on developing new materials that are eco-friendly, sustainable, and long-lasting with low energy consumption, superior quality, and lower production costs to replace Portland cement in some percentage have been conducted [16,17]. The use of Silica fume in PCPC enhanced water permeability [18] and also effectively increased early-age strength [19]. Fly ash in concrete could promote micro-damage on the phase boundary [20]. It was stated that the addition of ground granulated blast-furnace slag (GGBS) in concrete recused the porosity [21]. The usage of pozzolanic materials, such as silica fume, fly ash, and GGBS, in the concrete industry is limited. For many years, natural pozzolans have been used to manufacture lime-pozzolanic mortars [22]. When cement, lime, and water are mixed with naturally occurring pozzolanic resources, such as siliceous or aluminous materials or silicious materials, cementitious compounds are produced. Using natural pozzolanic materials such as volcanic tuff, diatomaceous earth, perlite, and pumice as partial substitutes for Portland cement material may result in energy and cost savings [23]. The strength development of concrete with a high volume of SCMs is normally 30% lower than plain cement concrete on the 28th day [24].

Diatomaceous earth (DE) is reported to exhibit pozzolanic activity when blended with cement [25,26]. DE is highly porous in nature, with the void content ranging between 80 and 90% [21]. The amount of silica content of DE may vary from 86 to 94% of weight [27]. DE can be used as filler, adsorbent, insulator, etc. [28]. Cement can be replaced by DE, fly ash (FA), and limestone to reduce the negative impact on the environment while producing mortars. [29]. In the US, the cost of DE used as a lightweight aggregate and binder averages USD 10/tonne or 9% of the cost of Portland cement [30]. However, recycled DE can also be integrated into concrete. DE utilized in the agroindustry, brewery, and distillery industries is disposed of by landfill [31]. However, DE is not frequently used in the construction industry because of its high water demand [32]. Diatomite deposits are primarily made up of opal diatom skeletons or amorphous silica, where the dominant diatom species differs between deposits [33]. Very dense and long-lasting concrete can be created by reducing the water/cement ratio (w/c ratio) to less than 0.3 by mass and adding amorphous silica [34,35]. Mixtures containing DE show strength improvements, exhibit early-age properties, and are more suitable for general applications [36]. Silica contributes to an increase in the properties of concrete by double-fold by inducing C-S-H formation to a larger extent via pozzolanic reaction and acts as a filler to fill the voids between cement and other compounds [37]. The microstructure matrix of concrete-incorporated DE was refined because of the lime-silica reaction, and the interfacial transition zone (ITZ) was denser; therefore, the microstructural changes could enhance the durability properties of cementitious composites containing DE as SCMs [38].

Previous studies mainly focused on illustrating and knowing the functional as well as structural properties of PCPC; however, very limited studies evaluated the effects of natural silica sources as supplementary cementitious materials for PCPC application. On the other hand, although the application of DE in concrete and building materials is not a new topic, there are very few studies on the use of DE for Portland cement porous concrete application. The main objective of this study is to improve the understanding of the effect of DE in pervious concrete. In this study, the cement was partially replaced by DE as an SCM. A series of experimental tests, such as consistency and water demand, compressive strength, split tensile strength, flexural strength, void content, and water permeability tests, were conducted on the samples.

## 2. Materials and Methods

### 2.1. Materials

The production of PCPC includes cement, coarse aggregate, water, and diatomaceous earth as its primary constituents. In this study, the addition of a small fine aggregate (10%) was applied to the mix in terms of improving the strength of the concrete. In addition, the cement was replaced by various amounts of DE.

#### 2.1.1. Aggregates

A single source crushed rock having an average particle size of 19 mm was used in this research. The typical grading of coarse aggregate used in PCPC conforms to the specifications of ASTM C33/C33M [39]. Accordingly, 64% and 96% of aggregates were retained with 12.5 mm and 4.75 mm sieves, respectively. The conventional river sand was used as fine aggregates. The particle size distribution and fineness modulus were examined as per ASTM C33, and accordingly, 92% of sand was passed on a 2.36 mm sieve, and those which were retained on the 4.75 mm sieve were cast off. The different properties of aggregates are represented in Table 1.

#### 2.1.2. Cement

**Figure 1:**
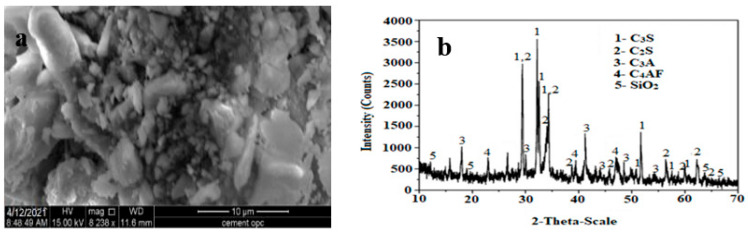
(**a**) SEM image of OPC and (**b**) XRD image of OPC.

The cement used in this study was commercial ordinary Portland cement (OPC) with the market branded as Coramantal and conformed to ASTM C150/C150M-22, 43 N, [40], with a density of 3.15 g/cm^3^. Using a JEOL-JSM-IT 200, equipped with EDS, the scanning electron microscopy (SEM) test was carried out. SEM test specimens, in powder forms, were put on platinum-coated carbon adhesive tape and were subjected to characterization examination utilizing a voltage of 15 kV in a high vacuum camper. The phase composition of cement was characterized using the X-ray diffraction (XRD) analysis. The sample was prepared in powder form, put inside a sample holder, and mounted on a triple-axis (Ge-220) monochromatic detector. At room temperature, the sample analysis was carried out utilizing 45 kV, 40 mA, with 2 exploration range. The scanning electron microscope (SEM) and X-ray diffraction (XRD) images of cement are shown in Figure 1a,b, respectively. Cement exhibits amorphous quality with bulky structure in SEM image; meanwhile, XRD humps with high narrow peaks exhibit it as having a crystalline nature with Alite, Belite, Tricalcium aluminate, and Tetracalciumaluminoferrite. The chemical combination of OPC is represented in Table 2.

#### 2.1.3. Water

Clean tap water was employed for material washing and other experimental purposes. The water quality used in this study was drinkable, as discussed in ACI 301 [41]. The ratio of water to binder was maintained based on the standard consistency, and water content was carefully regulated.

#### 2.1.4. Diatomaceous Earth

**Figure 2:**
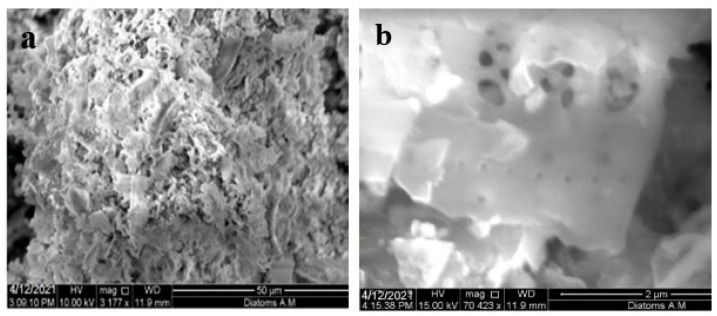
(**a**) SEM image of DE at 50 µm and (**b**) SEM image of DE at 2 µm.

The DE samples were collected from Jaipur, Rajasthan, India. After one day of natural surface drying, the obtained DE sample was crushed and sieved by using a sieve size of 200 μ. Following that, the DE powder was dried in the oven for 24 h at 100 °C. The DE particles showed a bulk density of 775 kg/m^3^. The specific gravity of DE in standard drying conditions was obtained at 2.06, while it was evaluated at 1.92 after oven drying with absorption of 6.71%. The SEM images of DE at 50 µm and 2 µm are represented in Figure 2a,b, respectively. DE exhibits a tubular microstructure with nanopores and tube walls. As can be seen from Figure 2b, the pores are empty with loose nature. A similar kind of nature was already recorded in previous research [33]. The chemical composition of DE is described in Table 3.

### 2.2. Methods and Mix Proportion

To find reliable mean values, triplicate specimens were prepared for each test group. The samples were prepared as per ASTM C1688/C1688M [42]. The mix proportion was prepared based on ACI 522 R-10 [43] and proportioning phase of the NRMCA Portland cement mixture (National Ready Mix Concrete Association). In order to produce the control mix, 10% of fine aggregate and OPC were added to the place of the coarse aggregate. For the desired porosity of 15%, a control mix with w/b of 0.28 and paste content of 0.27 m^3^ was used.

The total volume of solids, including the aggregates, water, and cementitious materials, was estimated to be 0.85 m^3^. Cement was partially replaced by DE by weight at various contents of 5%, 10%, 15%, 20%, and 25%. The different mix ratios are given in Table 4. The freshly prepared concretes were cast in cube mold specimens of 100 mm × 100 mm × 100 mm, cylinders of 100 mm × 300 mm, and beams of 100 mm × 100 mm × 500 mm. Three identical layers of fresh concrete in steel modules were compacted manually with sixty strokes each, followed by ten seconds of compaction on a mechanical vibration table. The specimens and their molds were covered by a plastic cover and allowed to cure at room temperature of 24 ± 1 °C for 24 h. After that, the molds were removed, and specimens were allowed for 27 days of water curing.

The compressive strength test was performed at the age of 7 days and 28 days by following the ASTM C39 [44] testing method. Splitting tensile strength and flexural strength tests were also conducted on the samples cured for 7 days and 28 days following ASTM C496-96 [45] and ASTM C78/C78M-22 [46], respectively. The volume displacement method was used to determine the amount of void content in each cylindrical sample at the age of 28 days. The dry mass and the underwater mass of concrete samples were measured according to ASTM C1754/C1754M-12 [47]. The permeability coefficient of the PCPC specimens was determined using the water permeability test. The test was executed as per ASTM C1701/1701M [48]. The infiltration test was executed after the concrete specimens were covered with rubber tabs to avoid the water flow outside the specimen. In this test, the time period (sec) required for the water to enter the specimen was recorded, and the permeability coefficient was determined using the formula shown below.

$$I=\frac{KM}{D^{2}\times t}$$

where *I* = ratio of infiltration (mm/h), *M* = weight of infiltrated water (kg), *D* = infiltration ring inner diameter (mm), *t* = time taken for the measured volume of water to infiltrate the concrete (s), and *K* is 4,583,666,000 ((mm^3^ s)/(kg·h)).

## 3. Results and Discussion

### 3.1. Characteristics of Cement and DE

The DE showed a cluster of irregular and angular particles with brushy organics. When observed in the 2 µm range, DE showed thin, angular particles with blade-type crystals along with a high amount of inner porosity. The phase composition of OPC was analyzed using XRD. The OPC was found to contain different calcium compounds: C_2_S, C_3_S, and C_3_A (expressed as CaO, it was 69%, Table 2), whereas the proportion of silica was almost three times that of alumina, and ferrous occupied almost half the percentage of alumina. In DE, silicon dioxide was obtained at 82%, which is an important property that is attributed to cement hydration. This enormous amount of silica can react with Ca(OH)_2_ and water to produce C-S-H gel, which acts as the primary binder of cement concrete. Moreover, oxides of iron and calcium made up 2% and less than 0.5%, respectively.

### 3.2. Consistency and Water Demand

**Figure 3:**
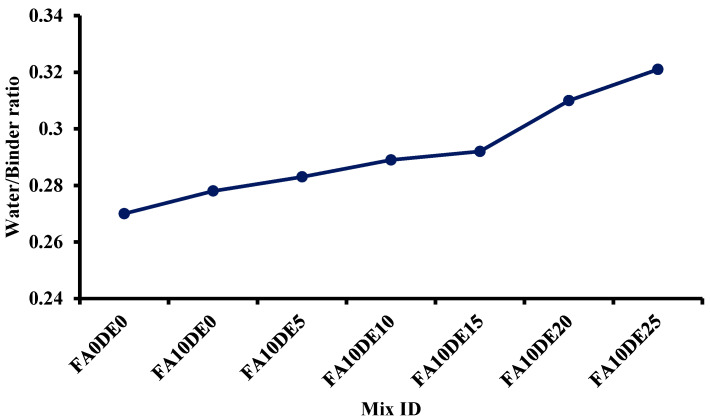
Consistency test for water demand.

The addition of DE led to an increment in water demand as DE has a high amount of inner porosity, which is consistent with the SEM analysis. The mix design was adjusted based on the standard consistency test result for control and each level of replacement. The control cement paste achieved its consistency at the w/b of 0.28, while cement partially replaced by 25% DE achieved its standard consistency at the w/b of 0.32. The design mix was adjusted according to the water demand of each level of replacement, as it plays a key role throughout the hydration process as well as physical properties. Figure 3 shows the water demand at each level of replacement.

### 3.3. Compressive Strength Test

**Figure 4:**
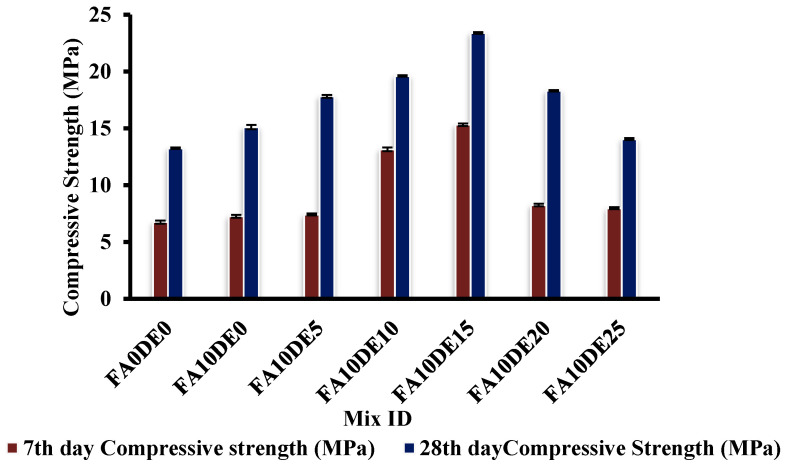
Compressive strength of various replacements.

The results are shown in Figure 4. The load was applied at a rate of 6.2 kN/s. The average compressive strength of the PC on the 28th day was 15.15 MPa. The PCPC, at a replacement level of 15% by weight of cement, attained an average compressive strength of 23.40 MPa, demonstrating an increase of 54.45% compared to the control mix. The obtained results can be due to the presence of amorphous silica in DE that might form calcium hydrate silicate by reacting with Ca(OH)_2_ and water, leading to increased concrete strength [49]. The results are consistent with those provided by Degimenci and Yilmaz [50]. They claimed that the rate of increase in the compressive strength of mortars containing diatomite relies on the level of cement replacement [50]. Beyond 15%, there is a decrease in compressive strength that may be caused by an inadequate amount of calcium hydroxide (C-H) in blended cement, which would be needed to react with much more silica following DE addition [51]. It was also reported that mechanical parameters could be improved by up to 15% substitute for Portland cement [52]. The design shows that partially replacing cement with DE by up to 20% enhanced the compressive strength compared to the control mix.

### 3.4. Split Tensile Strength

**Figure 5:**
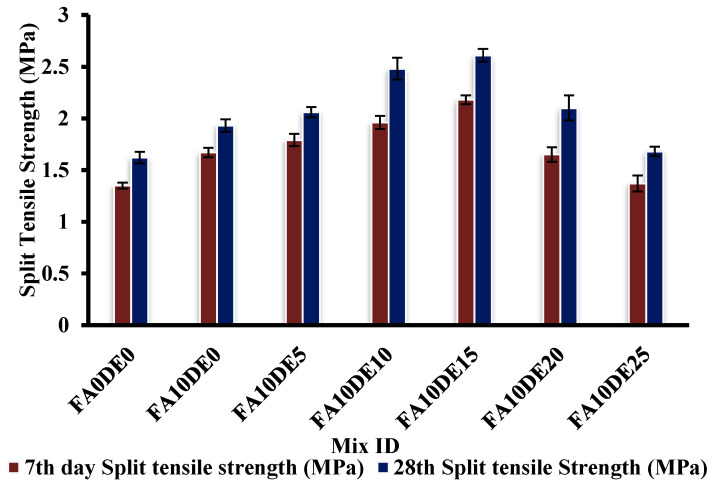
Split tensile strength of various replacements.

The average split tensile strength of PCPC specimens on days 7 and 28 is shown in Figure 5. It also exhibited the same trend as the compressive strength test. On the 28th day, the control specimen recorded a split tensile strength of 1.39 MPa; however, the split tensile strength of the sample FA10DE15 was 36.84% higher than the control specimen. This can be because of the high amount of C_3_S and C_2_S in cement that produces Ca(OH)_2_, while reacting with water, which reacts with alumina and amorphous silica in DE to generate an excessive amount of C-S-H. The excess amount of C-S-H can replace the pore holes in the concrete mix and refine and densify the interfacial transition zone (ITZ) between aggregate particles and cement paste [53]. The immediate filler effect of DE was also reported by [52]. Moreover, a similar kind of split tensile strength increment at an early-age strength increment was recorded by [54]. Similarly, the average split tensile strength of the sample FA10DE20 was found to be 10.52% higher than the control mix. However, a 25% replacement level showed an 11.76% strength decrement to the control mix. Although replacement beyond 15% is not considered a reduction in cement particles, that could lead to the possibility of less hydrated product availability, resulting in the reduction of tensile strength. Similar results of the reduction in tensile strength were also reported by [55].

### 3.5. Flexural Strength

**Figure 6:**
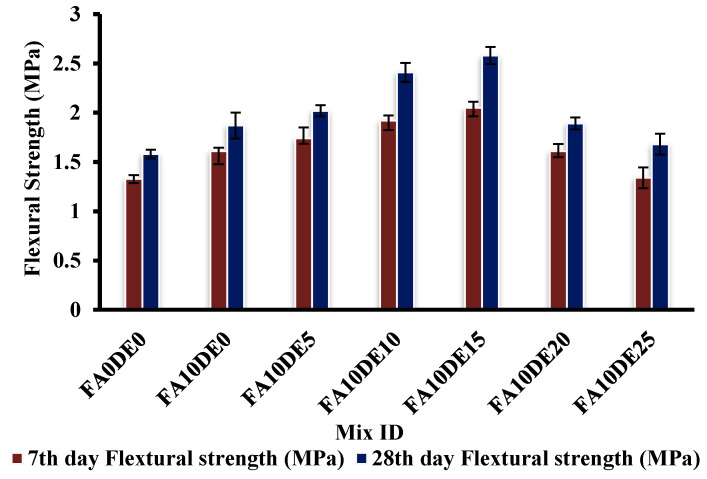
Flexural strength of various replacements.

Figure 6 shows the flexural test results of PCPC specimens. In the 28th day test, the average flexural stress value of the control specimen was found to be 1.35 MPa, whereas the sample FA10DE15 achieved a flexural strength of 2.55 MPa, indicating an enhancement of 37.84% in the flexural strength compared to the control mix. When compared to the control mix, it was found that the flexural strength values increased by up to 20%. The increase in flexural strength could be attributed to the enhancement and densification of the ITZ. In addition, a direct relationship between the adhesion of aggregates with paste that occurs because of the excess hydrated products from the pozzolanic reactions between SiO_2_ and Ca(OH)_2_ contributed to the bending resistance, thereby increasing the flexural strength [56]. Saidi et al. proved that the addition of DE in concrete enhanced flexural strength at early ages [52]. However, beyond 15%, a decline in flexural strength was observed, which could be due to the agglomeration of amorphous silica and high water absorption that led to a reduction in consistency.

### 3.6. Void Content of PCPC

As mentioned previously, the volume displacement method was used to determine the amount of void content in each cylindrical sample at the age of 28 days. Table 5 demonstrates the porosity of pervious concrete samples. The experimental results indicate that the partial replacement of DE significantly reduced porosity. In the absence of DE, the control mix exhibited 26% of porosity. However, when cement is replaced by 15% DE, 15.4% porosity was identified. The reduction in porosity could be because of the presence of amorphous silica in DE that reacted with Ca(OH)_2_ and caused C-S-H gel formation, which helped to fill the pores in the concrete and made the PCPC denser than the control concrete. The addition of DE in cement concrete could reduce the voids. This result is similar to the previous findings of [53]. However, the desired porosity of PCPC was not achieved beyond 15% replacement. The amount of void content was determined using the following formula:

$$\mathrm{VCR}=\left(1-\left[\frac{K\times \left(A-B\right)}{\rho_{w\;}\times D^{2}\times L}\right]\right)\times 100$$

where VCR = void content ratio (%), *K* = 1,273,240 ((mm^3^·kg)/(m^3^·g)), *A*= weight of dry specimen (g), *B* = weight of specimen underwater (g), ρw = experimental water density (kg/m^3^), *D* = average specimen diameter (mm), and *L* = average specimen length (mm).

### 3.7. Water Permeability Test

The values of the permeability coefficient are presented in Table 6. The results exhibit that the permeability coefficient lies between 14.2 mm/s and 4.7 mm/s indicating that the addition of DE effectively decreased the pores and hydraulic conductivity. In fact, the reaction of amorphous silica and cement formed a coat around aggregates. This coating can also reduce the penetration of water molecules.

### 3.8. Young’s Modulus vs. Compressive Strength

**Figure 7:**
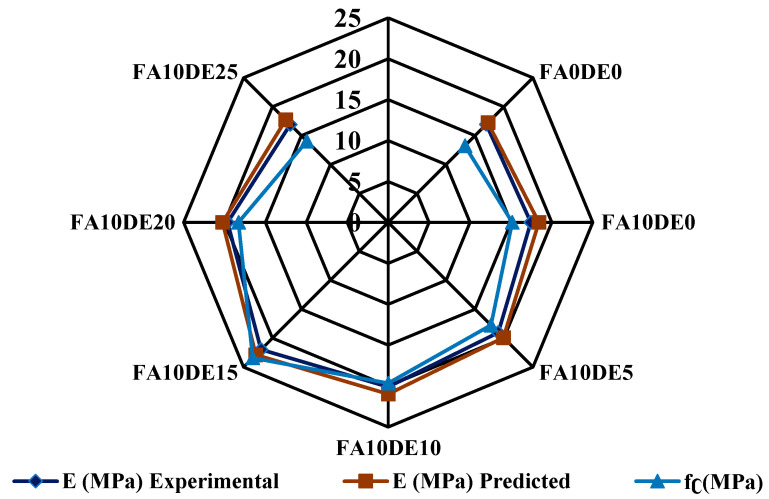
Relation between Young’s modulus and compressive strength.

Figure 7 shows the relationship between the compressive strength and Young’s modulus of the samples. The predicted values were determined according to ACI 318 [57] as mentioned below. The experimental result shows that the addition of DE improved Young’s modulus of PCPC, indicating that DE densified the inner matrix between aggregates and cement paste. However, the addition of a large amount of DE showed a negative impact due to the generation of an inadequate amount of C-H to react with all the available silica leaving some amount of silica without any chemical reaction, which in turn acted as a filler material between the inner matrixes of PCPC. The calculated and experimental Young’s modulus values are almost similar for 10 to 20% cement replacement with DE.

$$E_{c}=4730\sqrt{F_{c}}$$

where E_c_ = Young’s modulus of concrete, and *F_c_* = compressive strength of concrete.

### 3.9. Statistical and Regression Analysis

**Figure 8:**
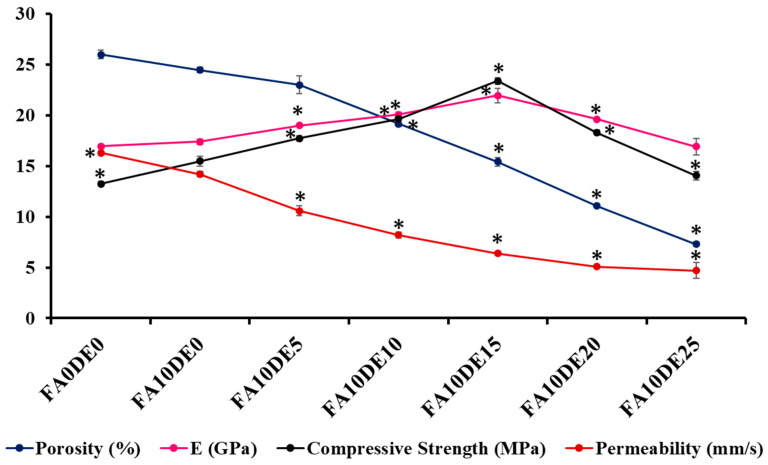
ANOVA results; the significance of various physical properties compared with the replacement of cement with DE in different concentrations. * indicates significance at *p* < 0.05 when compared between FA10DE0 and other mix design groups.

**Figure 9:**
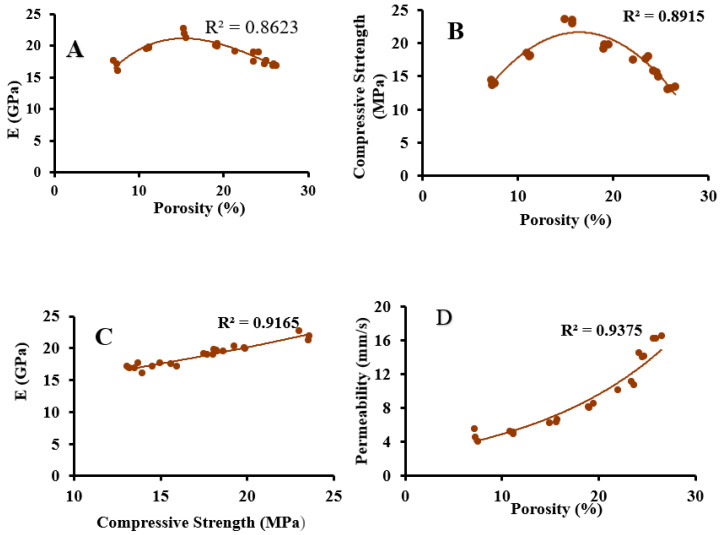
Scatter plots for different physical properties of PCPC. (**A**) Young’s Modulus vs. Porosity; (**B**) Compressive strength vs. Porosity; (**C**) Young’s Modulus vs. Compressive strength; and (**D**) Permeability vs. Porosity.

One-way ANOVA (SPSS 17) was used to find the significance between the control mix and various replacement mixtures. Further, regression analysis was performed to define the relationship between various physical features of PCPC, and the goodness of fit was represented as scatter plots (Figure 8). The results of ANOVA indicate that there is a significant change (*p* < 0.05) in the physical properties of concrete upon the addition of DE, and the optimum concentration of cement to be replaced by DE was found to be 15% (Figure 8). Figure 9A shows that the addition of DE decreases the porosity while increasing Young’s modulus up to the optimum level (15% cement replacement by DE). Beyond optimum, it acts like filler material without any bond between the aggregates. Similarly, compressive strength also shows the same trend in relation to porosity, with the reduction in strength contradicting the general view beyond optimum (Figure 9B). This may be because compressive strength, in addition to porosity, also depends on other factors, including the amount of C-H produced during the hydration of cement as well as bond strength. Further, the compressive strength depicted a progressive linear relation with Young’s modulus (Figure 9C). Finally, permeability showed a reduction with a decrease in porosity (Figure 9D) with the addition of DE because the amorphous silica present in DE effectively arrests the water penetration.

## 4. Conclusions

Although several studies explored the potential use of DE in concrete, there are very limited works on the use of DE for Portland cement porous concrete application. Therefore, the aim of this research was to evaluate the performance of PCPC having cement partially replaced by DE. The study results can be summarized as follows:

- The diatomaceous earth was observed to be a lightweight SCM having soft minerals with a rich amount of amorphous silica. The SEM image showed that the diatomaceous earth particles have high porosity.
- It was observed that the water demand gradually increased up to the optimum inclusion of DE, but the higher replacement of cement with DE resulted in a drastic increment in water demand.
- The strength of pervious concrete containing diatomaceous earth increased in all replacements. The 25% replacement showed only a slightly higher strength than the control mix on the 28th day. However, the 15% replacement, the optimum content, exhibited 54.45%, 36.84%, and 37.84% higher compressive, split tensile, and flexural strengths, respectively, compared to the control mix.
- The correlation between porosity and permeability showed an inverse relation upon increasing diatomaceous earth concentration. The desired porosity of 15.42% was achieved in 15% replacement. The relationships between Young’s modulus and compressive strength with both experimental and predicted values by partial replacement of cement by diatomaceous earth were almost similar within the range of 10–15%.
- The addition of diatomaceous earth as an SCM in cement would decrease the amount of cement consumption, contributing to the reduction of carbon footprint in the atmosphere.

## Figures and Tables

**Table 1:** Properties of aggregates.

Sl. No	Properties	Coarse Aggregate	Fine Aggregate
1	Specific gravity	2.62	2.50
2	Bulk density loose (g/cm^3^)	1.48	1.46
3	Fineness modulus	3.7	2.77
4	Water absorption (%)	1.3	Nil
5	Abrasion mass loss (%)	20	-
6	Silt content (%)	-	4.4

**Table 2:** Chemical constituents of OPC.

Compound	SiO_2_	Al_2_O_3_	Fe_2_O_3_	CaO	Na_2_O	K_2_O	TiO_2_	MgO	SO_3_	LoI
% in mass	18	6.4	2.8	69	0.46	0.79	1.53	0.89	1.64	1.28

**Table 3:** Chemical constituents of DE.

Compound	SiO_2_	Al_2_O_3_	Fe_2_O_3_	CaO	MgO	Na_2_O	K_2_O	MnO	P_2_O_5_	TiO_2_	H_2_O	LOI
% in mass	82.00	<0.01	2.00	0.22	0.30	0.10	0.20	0.06	0.34	0.19	1.15	14.18

**Table 4:** Mix proportions of PC.

Mix ID	% of Replacement	Design Porosity (%)	Cement (kg/m^3^)	Water (kg/m^3^)	Coarse Aggregate (CA) (kg/m^3^)	Fine Aggregate (FA) (kg/m^3^)	DE (kg/m^3^)
FA0DE 0	0	15	460.86	124.73	1453.78	0	0
FA10DE 0	0	15	402.96	112.02	1313.09	139.69	0
FA10DE 5	5	15	382.81	114.04	1313.09	139.69	20.15
FA10DE 10	10	15	362.66	116.45	1313.09	139.69	40.30
FA10DE 15	15	15	342.52	117.66	1313.09	139.69	60.44
FA10DE 20	20	15	322.36	124.92	1313.09	139.69	80.60
FA10DE 25	25	15	302.22	133.82	1313.09	139.69	100.74

**Table 5:** Porosity test result.

Sl.No	Mix Id	Design Porosity (%)	Actual Porosity (%)
1	FA0DE0	15	26.02
2	FA10DE0	15	24.47
3	FA10DE5	15	23.02
4	FA10DE10	15	19.17
5	FA10DE15	15	15.42
6	FA10DE20	15	11.08
7	FA10DE25	15	7.31

**Table 6:** Permeability Coefficient.

Sl.No	Mix Id	Permeability Coefficient (mm/s)
1	FA0DE0	16.3
2	FA10DE0	14.2
3	FA10DE5	10.6
4	FA10DE10	8.2
5	FA10DE15	6.4
6	FA10DE20	5.1
7	FA10DE25	4.7

## Data Availability

Not applicable.
